# Learning Curve of Da Vinci Xi Robotic Low Anterior Resection: A Cumulative Sum Analysis of a Single High-Volume Surgeon

**DOI:** 10.3390/jcm15031248

**Published:** 2026-02-04

**Authors:** Yu-Kang Tseng, Feng-Fan Chiang, Ming-Cheng Chen, Chun-Yu Lin

**Affiliations:** Division of Colorectal Surgery, Department of Surgery, Taichung Veterans General Hospital, Taichung 407, Taiwan; 10510ken@gmail.com (Y.-K.T.);

**Keywords:** rectal cancer, robotic surgery, low anterior resection, da Vinci Xi, learning curve, CUSUM

## Abstract

**Background:** The learning curve for robotic low anterior resection (LAR) utilizing the modern da Vinci Xi system within a high-volume, standardized environment remains poorly defined. This study aimed to delineate the technical proficiency of a single high-volume surgeon using the Xi platform. **Methods:** A retrospective analysis of 95 consecutive patients undergoing robotic LAR for primary rectal malignancy between 2020 and 2023 was conducted. All procedures were performed by a single surgeon using the da Vinci Xi system under a standardized ERAS protocol. Cumulative sum (CUSUM) analysis of operative time was used to define learning phases. **Results:** CUSUM analysis identified a proficiency inflection point after 16 cases. Median docking time significantly decreased in the proficiency phase (14.5 vs. 10.0 min, *p* < 0.01). Notably, zero conversions to open surgery occurred throughout the series. Comparative analysis revealed comparable overall complication rates (0.0% vs. 13.9%, *p* = 0.201) and postoperative length of stay between phases. Short-term oncological quality, including lymph node yield and circumferential resection margins, remained satisfactory in both groups. Technical precision, reflected by consistently low robotic stapler firings (median 2.0), was maintained from the outset. **Conclusions:** Technical proficiency in robotic LAR using the da Vinci Xi system was rapidly achieved after approximately 16 cases in this high-volume standardized setting. This accelerated learning curve was not associated with compromised perioperative safety or oncological outcomes.

## 1. Introduction

Taichung Veterans General Hospital (TCVGH) stands as a premier tertiary referral center in central Taiwan, distinguished by a two-decade legacy of leadership in minimally invasive surgery. The institution has consistently pioneered the adoption of advanced surgical technologies. The da Vinci surgical system was first introduced at TCVGH in November 2005, marking a significant milestone in regional surgical care. By mid-2024, a cumulative total of over 6000 robotic procedures across various specialties had been completed, underscoring the institution’s profound experience. Recognition of this expertise is exemplified by TCVGH’s designation as Taiwan’s only “Total Program Observation” (TPO) center, hosting four certified robotic surgical demonstration specialties, including the Division of Colorectal Surgery [1]. As a last-line referral institution, TCVGH frequently manages complex, locally advanced rectal cancer cases transferred due to high surgical difficulty.

The Division of Colorectal Surgery at TCVGH has a longstanding tradition of performing laparoscopic total mesorectal excision (TME), which has been the standard of care for many years [2]. Building upon this solid foundation in minimally invasive techniques, the robotic colorectal program was inaugurated in 2018, initially utilizing the da Vinci Si system [3]. To ensure the highest degree of technical consistency and to leverage the latest technological advancements, the present investigation exclusively focuses on procedures performed using the da Vinci Xi platform (Intuitive Surgical, Sunnyvale, CA, USA). The Xi system became the standard platform for rectal cancer surgery at the institution from 2019 onwards, offering enhanced capabilities such as multi-quadrant access and integrated table motion.

A hallmark of the surgical approach at TCVGH is the rigorous standardization of perioperative care. All patients undergoing colorectal procedures, including robotic low anterior resection (LAR), are managed under a well-established Enhanced Recovery After Surgery (ERAS) protocol designed to optimize postoperative outcomes and accelerate functional recovery [3]. The landscape of robotic rectal surgery in Taiwan witnessed a pivotal shift on 1 March 2023, when the National Health Insurance (NHI) began covering the surgical fee component for robotic LAR [4]. While patients remain responsible for the costs associated with robotic instruments and accessories, this policy change has significantly increased accessibility to robotic surgery.

Despite the established benefits of robotic surgery for rectal cancer, the learning curve for mastering this complex procedure on the modern da Vinci Xi platform, particularly within a high-volume Asian center operating under a mature ERAS program, remains under-characterized. Most existing learning curve studies are based on older robotic systems or heterogeneous datasets [5,6,7]. Therefore, this study aims to precisely delineate the learning curve of robotic LAR using cumulative sum (CUSUM) analysis, based on the experience of a single high-volume surgeon exclusively utilizing the da Vinci Xi system at TCVGH, a designated robotic Mentor Site [8,9]. The investigation seeks to provide contemporary evidence on technical proficiency acquisition, perioperative safety, and oncological quality in the current era of widespread robotic adoption and evolving reimbursement models.

## 2. Materials and Methods

### 2.1. Study Design and Ethics

A retrospective observational study was conducted at a single tertiary referral center, TCVGH. The study protocol was reviewed and approved by the Institutional Review Board of TCVGH (IRB No. CE251051C). The requirement for informed consent was waived by the IRB due to the retrospective nature of the medical record review and the anonymization of patient data.

### 2.2. Study Population

To establish a highly homogenous study cohort reflecting contemporary robotic practice, medical records of consecutive patients who underwent robotic-assisted surgery for primary rectal malignancy between June 2020 and May 2023 were reviewed. Strict inclusion criteria were applied to ensure consistency. Only patients with tumors located anatomically in the rectum were included; cases classified in the surgical schedule as “robotic anterior resection” (AR) involving the sigmoid colon were specifically excluded.

To minimize confounding variables related to surgical complexity and protocol adherence, the following exclusion criteria were strictly applied: (1) emergency procedures, including those presenting with bowel obstruction or perforation; (2) combined surgeries requiring multi-organ resection; (3) benign tumors; and (4) patients who were not managed under the standardized ERAS protocol. Further exclusion criteria encompassed diagnoses of non-adenocarcinoma pathologies, carcinoma in situ (pTis), locally advanced tumors invading adjacent structures (pT4b), distant metastasis (Stage IV) at presentation, synchronous colorectal tumors, procedures utilizing the older da Vinci Si system, and operations performed by surgeons other than the principal investigator.

### 2.3. Surgeon Expertise and Standardization

A defining characteristic of this investigation is the rigorous standardization of the surgical environment and personnel. All interventions were performed exclusively by a single, highly experienced colorectal surgeon, who is recognized as a high-volume robotic surgeon according to international standards, thereby ensuring technical uniformity and baseline quality assurance throughout the study period [10,11,12]. The da Vinci Xi surgical system (Intuitive Surgical, Sunnyvale, CA, USA) was utilized exclusively for all procedures. Furthermore, perioperative care pathways were strictly standardized for all patients according to an established ERAS protocol previously detailed by this institution [3].

The operating surgeon is a high-volume expert with 20 years of laparoscopic experience (>2000 cases). Prior to adopting the da Vinci Xi system, his robotic experience was limited to 10 cases (6 LAR, 4 AR) performed on the da Vinci Si system (2018–2020). During the study period (2020–2023), the surgeon concurrently performed 140 other robotic procedures (including AR and hemicolectomies). This concurrent volume contributed to general platform familiarity, allowing the surgeon to focus on the specific technical demands of robotic TME in the narrow pelvis, which is reflected in the learning curve analysis.

### 2.4. Surgical Technique

A uniform surgical technique was maintained across all cases. Patients were placed in the standard lithotomy position requiring a steep Trendelenburg tilt and right-side rotation. Abdominal access was uniformly achieved via an open Hasson technique at the para-umbilical site for the initial camera trocar. The consistent four-arm port configuration and specific assistant port placement are illustrated in Supplementary Figure S1. Instrument assignment remained constant: Arm 1 utilized Fenestrated Bipolar Forceps (Intuitive Surgical, Sunnyvale, CA, USA) Arm 2 carried the da Vinci Xi Endoscope (Intuitive Surgical, Sunnyvale, CA, USA); Arm 3 (a 12-mm port) was designated for Monopolar Curved Scissors, replaceable by the da Vinci SureForm™ Stapler (Intuitive Surgical, Sunnyvale, CA, USA) as needed; and Arm 4 utilized a Vessel Sealer (Intuitive Surgical, Sunnyvale, CA, USA). A 5 mm assistant port was located on the patient’s right flank.

The procedure followed a standardized medial-to-lateral approach. High ligation of the inferior mesenteric vein (IMV) was performed first, followed by the inferior mesenteric artery (IMA). Sharp dissection was maintained strictly within the avascular “holy plane” to achieve TME principles [13]. The mesentery of the distal rectum was cleared (de-fatted) using the Vessel Sealer, and rectal transection was completed using the robotic SureForm™ Stapler. Mobilization of the splenic flexure was not routinely performed. Upon completion of the distal rectal transection, the robot was undocked. In adherence to the surgeon’s standardized protocol for this series, bowel continuity was restored via an extracorporeal anastomosis using an ECHELON CIRCULAR™ Powered Stapler (Ethicon, Cincinnati, OH, USA), with the size selected based on individual patient anatomy. Air leak testing was routinely conducted; however, intraoperative indocyanine green (ICG) fluorescence angiography was not part of the standard protocol [14].

### 2.5. Data Collection and Definitions

Clinical data were rigorously abstracted and cross-verified using three distinct sources: physician electronic medical records, operative nursing logs, and anesthesia reports to ensure maximum accuracy. Key operative timing metrics were stringently defined to reflect specific phases of the surgical workflow [15]. Docking time was defined as the interval commencing from the positioning of the patient cart adjacent to the operating table (after completion of port placement) and concluding when all robotic arms were correctly coupled to the trocars and ready for operation [16]. Due to the standardized extracorporeal anastomosis technique employed, console time was specifically defined as the duration from the surgeon taking control at the console until the completion of the distal rectal transection immediately prior to robot undocking. Intraoperative estimated blood loss (EBL) was recorded from anesthesia charts. It should be noted that a recorded value of 10 mL represents the minimum documentable unit at this institution, signifying nominal or non-quantifiable minimal bleeding.

The primary outcome measure for the learning curve analysis was defined as the console time. This metric was selected to specifically isolate the surgeon’s technical adaptation to the robotic interface, minimizing the influence of team-dependent variables such as anesthesia induction or room setup. Secondary outcomes included efficiency metrics (total operative time and docking time) to assess the maturation of the multidisciplinary team, as well as perioperative safety and quality indicators, including estimated blood loss, conversion rates, postoperative complications, length of stay (LOS), and pathological outcomes (lymph node harvest and resection margins).

### 2.6. Statistical Analysis

All statistical analyses and data visualizations were performed using Python programming language (version 3.8; Python Software Foundation, Wilmington, DE, USA), utilizing standard scientific libraries including pandas for data manipulation, NumPy for numerical operations, and SciPy for statistical hypothesis testing [17]. Categorical variables are presented as frequencies and percentages, while continuous variables are expressed as means ± standard deviations (SD) for normally distributed data or medians [interquartile range (IQR)] for non-normally distributed data, as determined by the Shapiro–Wilk test. To precisely delineate the learning curve phases based on operative time, the CUSUM analysis method was employed. The CUSUM technique is chosen for its sensitivity in visualizing subtle deviations from the process mean over consecutive cases, making it highly effective for identifying inflection points representing transitions in technical proficiency [18]. To assess the robustness of the proficiency inflection point identified by CUSUM, a sensitivity check was performed using total operative time as an alternative metric. Furthermore, linear regression analysis was applied as a secondary validation method to visually confirm the structural break and change in slope between the identified learning and proficiency phases. Subsequent comparative analysis between the identified learning phases was conducted using independent *t*-tests or Mann–Whitney U tests for continuous variables, and Pearson’s chi-square tests or Fisher’s exact tests for categorical variables, as appropriate. A two-sided *p*-value of <0.05 was considered statistically significant.

## 3. Results

**Figure 1:**
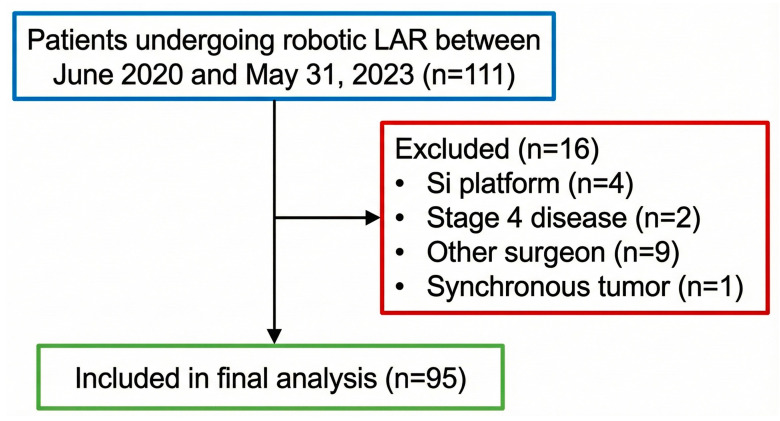
Flowchart of patient selection.

A total of 111 patients undergoing robotic LAR for rectal cancer between June 2020 and May 2023 were initially assessed for eligibility. Application of the exclusion criteria, as delineated in the study flowchart (Figure 1), resulted in the exclusion of 16 cases (comprising four da Vinci Si system procedures, two Stage IV cases, nine procedures performed by different surgeons, and one instance of synchronous tumors). Consequently, the final analytical cohort consisted of 95 consecutive patients operated on by a single surgeon using the da Vinci Xi platform.

Baseline clinicopathological characteristics of the entire cohort were stratified according to the identified learning phases, as summarized in Table 1. Statistical analysis revealed no significant disparities between the two groups regarding age, gender distribution, or body mass index (BMI). Furthermore, tumor characteristics, including the distance from the anal verge and Tumor–Node–Metastasis (TNM) stage distribution, were comparable between phases. Rates of neoadjuvant treatment and previous abdominal surgery also showed no statistically significant differences (all *p* > 0.05). These findings indicate that the patient population remained demographically and clinically homogenous throughout the study period. Among the 37 patients receiving neoadjuvant treatment, 23 underwent short-course radiotherapy (SCRT, 25 Gy in 5 fractions) and 14 received long-course concurrent chemoradiotherapy (CCRT, 50–50.4 Gy in 25–28 fractions). The specific chemotherapy regimens were tailored to clinical staging, primarily consisting of either FOLFOX (5-fluorouracil, leucovorin, and oxaliplatin) or oral Capecitabine.

**Figure 2:**
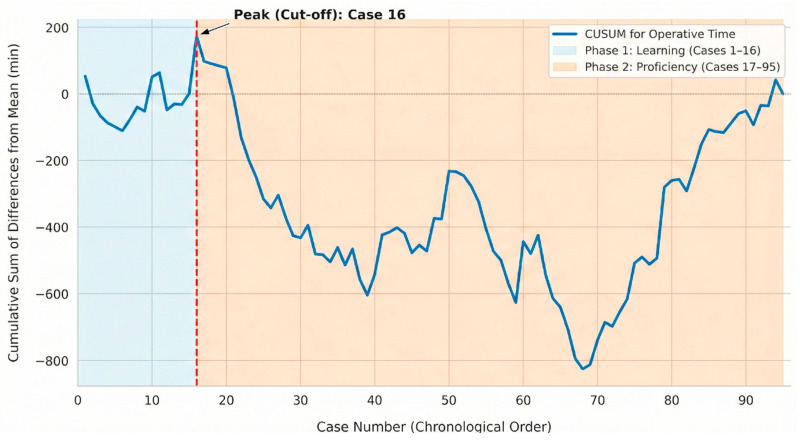
CUSUM analysis of operative time for 95 consecutive robotic LARs.

Learning Curve Assessment CUSUM analysis was utilized to define the learning curve based on operative time. The resulting CUSUM curve exhibited a distinct bell-shaped trajectory (Figure 2). The curve ascended initially, representing operative times exceeding the overall mean, until reaching its apex at case 16. This peak serves as the demarcating point for achieving technical proficiency. Accordingly, the initial 16 cases were categorized as the “learning phase” (Phase 1), while the subsequent 79 cases (cases 17–95) were designated as the “proficiency phase” (Phase 2).

**Figure 3:**
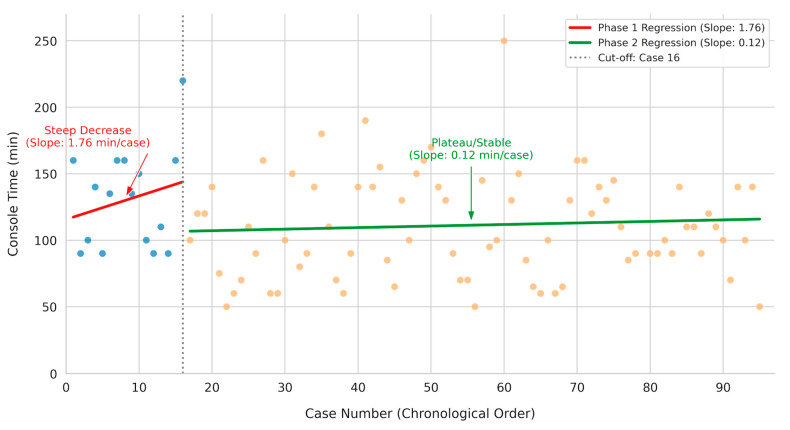
Scatter plot with linear regression analysis of console time versus case number. The blue dots represent cases in the learning phase (Phase 1: cases 1–16), while the orange dots represent cases in the proficiency phase (Phase 2: cases 17–95). The regression lines illustrate the trend of console time in each phase.

To further characterize the acquisition of technical skills, trends in console time were examined using linear regression modeling (Figure 3). The analysis revealed distinct patterns between the two phases. Phase 1 exhibited a slope of 1.76 min per case, indicating that operative times remained variable or slightly increased, likely reflecting the early introduction of complex cases. In contrast, Phase 2 demonstrated significant stabilization with a minimal slope of 0.12 min per case, confirming that technical proficiency had been reached and maintained despite case complexity, thereby providing visual corroboration of the learning phases identified by CUSUM analysis.

A detailed comparison of perioperative outcomes between the learning and proficiency phases is presented in Table 2. Regarding surgical duration metrics, the median console time decreased from 135.0 min in Phase 1 to 100.0 min in Phase 2 (*p* = 0.057). Similarly, median total operative times were statistically comparable between the two phases (327.0 vs. 315.0 min, *p* = 0.429). Technical stability in rectal transection was evident, as the median number of robotic stapler firings was identical between phases (2.0 vs. 2.0, *p* = 0.974), with a predominant use of 45 mm cartridges in both groups (100% vs. 87.3%, *p* = 0.204). Safety profiles remained robust; there were zero conversions to open surgery, and estimated blood loss was minimal (median 10 mL) in both phases.

**Figure 4:**
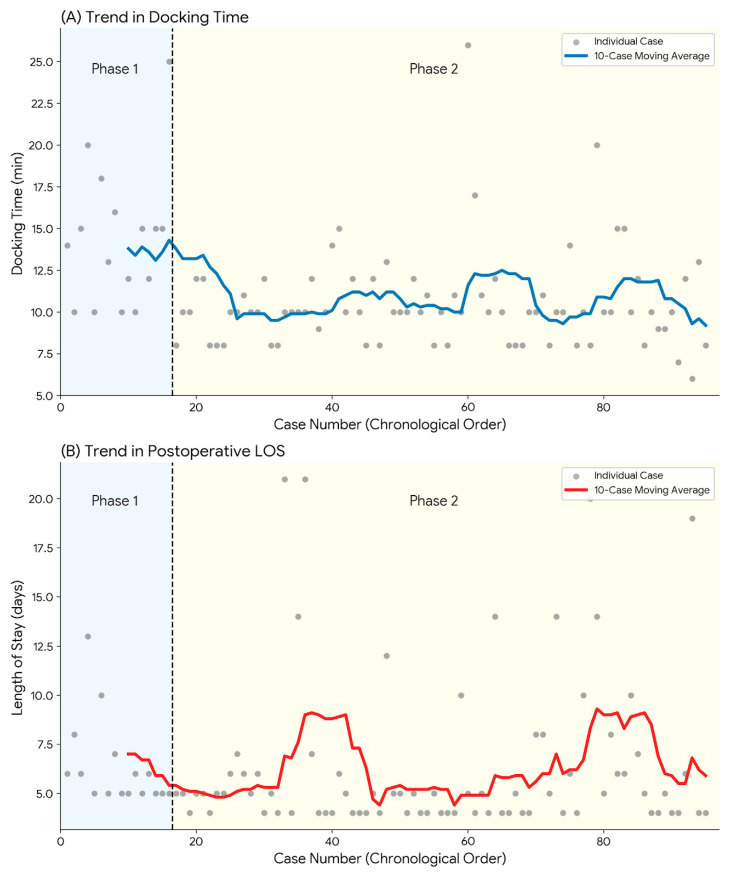
Chronological trends in perioperative outcomes across the learning curve. (**A**) Docking time. (**B**) Postoperative length of stay (LOS). The dashed lines represent the linear regression trend lines, indicating the change in outcomes over the case sequence.

The impact of accumulated experience on team efficiency was specifically highlighted by the changes in docking time. As shown in Table 2, there was a statistically significant reduction in median docking time from 14.5 min (IQR 11.5–15.2) in Phase 1 to 10.0 min (IQR 8.5–11.5) in Phase 2 (*p* < 0.001). This improvement is visually corroborated by the 10-case moving average trajectory in Figure 4A, which demonstrates a consistent downward trend, reflecting enhanced coordination with the Xi system setup among operating room personnel over time.

Postoperative recovery metrics demonstrated positive trends associated with increased proficiency. While the median time to soft diet remained comparable between phases (2.5 vs. 3.0 days, *p* = 0.459), the proficiency phase was associated with a statistically significant reduction in postoperative LOS (median 5.5 days [IQR 5.0–6.2] vs. 5.0 days [IQR 4.0–6.0], *p* = 0.036). This trend suggests that beyond mastering technical skills, the overall efficiency of postoperative care and patient recovery protocols continued to improve as the surgeon and team overcame the initial learning curve.

Pathological metrics reflecting the quality of oncological resection are summarized in Table 3. The oncological integrity of the procedures was maintained throughout the series. The median lymph node yield was high in both the learning phase (22.0 [IQR 18.5–27.2]) and the proficiency phase (20.0 [IQR 16.0–26.0]), with no statistically significant difference observed (*p* = 0.269). Rates of positive circumferential resection margin (CRM) < 1 mm were low in both groups (6.2% vs. 3.8%, *p* = 0.528). Notably, while a 100% negative distal margin rate was achieved in both phases, the median distal resection margin (DRM) was significantly longer in the proficiency phase compared to the learning phase (2.5 cm vs. 1.6 cm, *p* = 0.010). These results indicate that the quality of TME was established from the outset and was not compromised during the early learning curve.

## 4. Discussion

The present study successfully delineated the learning curve for robotic LAR utilizing the modern da Vinci Xi platform when performed by a single high-volume surgeon within a standardized ERAS environment. The results demonstrate a remarkably rapid acquisition of technical proficiency, with the inflection point observed after only 16 cases based on CUSUM analysis of operative time. Crucially, this accelerated learning phase was not associated with compromised perioperative safety or inferior short-term oncological specimen quality. These findings align with previous literature suggesting that high surgical volume [11] and prior laparoscopic experience [12] are critical determinants for shortening the learning curve, allowing robotic rectal surgery to be safely adopted by experienced colorectal surgeons.

The identification of a 16-case threshold for proficiency places the present series at the favorable end of the spectrum reported in contemporary literature. A recent systematic review analyzing learning curves in robotic colorectal surgery indicated a typical range of 15 to 30 cases to achieve stability [19]. Our findings align perfectly within this range but suggest faster acquisition than older studies predominantly based on the da Vinci Si system, where learning phases often exceeded 30–50 cases. This rapid progression may be partially attributed to the technological advancements of the Xi platform, such as integrated table motion and multi-quadrant access, which simplify complex workflows [6]. Furthermore, the influence of case volume and frequency cannot be overstated. In sharp contrast to a recent study from a community-based teaching institution reporting a prolonged learning phase extending up to 79 cases [20], the data presented here from a high-volume tertiary referral center suggests that concentrated surgical experience and a dedicated team significantly condense the learning trajectory.

Sensitivity analysis using total operative time yielded an identical inflection point (16 cases), confirming that the learning phase transition reflects both technical console proficiency and overall operative efficiency. However, console time was selected as the primary metric to specifically isolate the surgeon’s technical adaptation from team-dependent variables.

Beyond operative time, technical precision in rectal transection was evaluated as a surrogate for surgical mastery. The ability to achieve rectal division with fewer stapler firings is correlated with reduced anastomotic leak risks. In the current cohort, a low mean number of stapler firings (2.0, IQR 2.0–2.0) was achieved early in the learning experience and maintained throughout the series, with 45 mm cartridges predominantly utilized [21,22]. This metric underscores that critical technical skills required for safe TME dissection and transection were established from the outset, reinforcing the safety of the procedure during the initial phase. This observation aligns with findings from the ROLARR randomized clinical trial, which demonstrated that robotic capability regarding conversion rates is comparable to established laparoscopic standards even during eras of technological adoption [23]. Similar acceptable short-term outcomes have been reported in local comparisons of robotic versus laparoscopic approaches following neoadjuvant chemoradiotherapy (nCRT) in Taiwan [24].

An interesting observation in the comparison between phases was the divergence in perioperative outcomes. While complication rates did not show marked improvement in the proficiency phase (Phase 2) compared to the learning phase (Phase 1), the postoperative LOS significantly decreased (*p* = 0.036). The stability in complication rates despite faster operative times may be explained by a shift in case selection, often described as ‘complexity drift’ or a multiphasic learning process [25,26]. As technical confidence grew, the surgeon accepted increasingly complex cases [27]. Notably, detailed analysis of the final 15 cases in our series revealed that the proportion of patients who had undergone neoadjuvant treatment surged to 60.0% (compared to 31.3% in Phase 1), and those with prior abdominal surgical history increased to 26.7%. The tissue fibrosis associated with nCRT and adhesions from previous surgeries naturally necessitate more meticulous dissection. This phenomenon also elucidates the slight upward trend observed in the tail of the CUSUM curve. As described by Sng et al. [25], experienced surgeons in the later phases of the learning curve often undertake these technically demanding cases, where operative time naturally adjusts to accommodate increased difficulty, reflecting a prioritization of safety and precision over speed alone.

Regarding operative efficiency, the reduction in median console time from 135.0 min in Phase 1 to 100.0 min in Phase 2 showed a strong trend toward statistical significance (*p* = 0.057). Although marginally exceeding the traditional 0.05 threshold, this substantial 35 min reduction represents a clinically meaningful improvement in surgical proficiency. The lack of strict statistical significance likely reflects the limited statistical power of the learning phase sample (*n* = 16) combined with the aforementioned increase in case complexity during Phase 2. The ability to achieve faster median console times, despite operating on potentially more challenging patients, serves as a robust indicator that true technical mastery of the robotic platform has been achieved. The significant reduction in docking time (*p* < 0.001) observed in Phase 2 serves as a marker of multidisciplinary team maturation. As the nursing staff and surgical assistants became familiar with the specific docking angles and boom positioning of the Da Vinci Xi system, the setup process became more streamlined, independent of the surgeon’s console performance.

Furthermore, the exclusive utilization of extracorporeal anastomosis (ECA) in this series warrants specific context regarding operative efficiency and patient demographics. While intracorporeal anastomosis (ICA) is gaining traction globally, it is inherently associated with prolonged console times due to the complexity of robotic suturing and stapling [28]. Our adoption of a standardized ECA technique likely contributed to the favorable console times observed, as it bypasses the time-intensive intracorporeal reconstruction phase.

Moreover, this approach is particularly well-suited to our patient population. In contrast to Western cohorts where higher BMI and thickened mesentery often necessitate ICA to avoid tension during exteriorization, the relatively lower BMI of Asian patients allows for safe and easy specimen extraction [29]. Therefore, the ECA approach in this setting represents a time-efficient and anatomically appropriate strategy without compromising oncological safety.

Regarding methodology, CUSUM analysis was selected as the primary statistical tool due to its recognized sensitivity in detecting subtle shifts in performance over consecutive cases, representing a current gold standard in learning curve assessment. However, it is acknowledged that CUSUM peaks can be mathematically fluid and should be interpreted in conjunction with clinical outcomes rather than as absolute cut-offs [30]. Furthermore, while the single-surgeon design controls for operator variability, operative timings such as docking time and total operative duration are inevitably influenced by variations in the experience of assisting residents and circulating nursing staff, a factor inherent to the retrospective nature of the study. The timeline of this study aligns with the institutional introduction of the da Vinci Xi system in early 2020, prior to which the majority of procedures utilized the Si platform, as detailed in a previous report from the same surgical team [3].

Several limitations of this study must be acknowledged.

First, the study is inherently limited by its retrospective, single-surgeon design involving a senior consultant with extensive prior laparoscopic experience (>2000 cases). Consequently, the observed learning curve primarily reflects adaptation to the robotic interface rather than the acquisition of TME principles [31]. Without a comparative cohort of varying experience levels, we cannot empirically determine the specific laparoscopic caseload required to accelerate robotic proficiency. Therefore, our findings likely represent a “best-case scenario” and may not be fully generalizable to trainees or surgeons with limited laparoscopic background [32].

Second, potential selection biases cannot be entirely ruled out. Since the study was conducted during a period when robotic surgery required significant out-of-pocket expenses for instrumentation, the cohort may disproportionately represent patients with higher socioeconomic status [33]. This demographic often possesses better baseline nutritional status and health literacy, factors that could have contributed to more favorable perioperative outcomes compared to the general population, despite our attempts to adjust for comorbidities using ASA scores [34].

Third, regarding pathological assessment, while circumferential and distal margins were routinely reported, a standardized grading system for the completeness of the TME specimen was not routinely documented in pathology reports during the study period [35].

Fourth, the sample size of the learning phase (Phase 1, *n* = 16) is relatively small, which may limit the statistical power to detect differences in rare adverse events, such as anastomotic leakage or major complications (Clavien-Dindo ≥ III). Consequently, the lack of statistically significant differences in these safety outcomes should be interpreted with caution, as the study may be underpowered to identify subtle variations between phases.

Finally, the relatively short follow-up period precludes a robust analysis of long-term oncological outcomes such as disease-free and overall survival. However, the excellent short-term pathological outcomes observed in both phases provide confidence in the oncological safety of the procedure during the learning curve.

## 5. Conclusions

This study demonstrates that technical proficiency in robotic LAR utilizing the modern da Vinci Xi surgical system can be rapidly achieved by experienced colorectal surgeons, with a distinct stabilization of operative times observed after 16 cases. Notably, the transition to the proficiency phase was characterized not only by sustained safety but also by significant improvements in team efficiency, oncological quality, and patient recovery. These findings indicate that for surgeons with extensive laparoscopic background, the synergy of a high-volume environment and the advanced capabilities of the Xi platform significantly facilitate the acquisition of robotic skills, enabling a transition that is both efficient and oncologically superior.

## Figures and Tables

**Table 1:** Baseline Clinicopathological Characteristics.

Characteristic	Phase 1 (Learning) (*n* = 16)	Phase 2 (Proficiency) (*n* = 79)	*p*-Value
Age (years), mean ± SD	61.9 ± 14.3	61.8 ± 13.5	0.977
Gender (Male), *n* (%)	7 (43.8%)	47 (59.5%)	0.377
BMI (kg/m^2^), median (IQR)	22.8 (21.6–27.5)	23.3 (21.8–25.8)	0.676
ASA Score > 2, *n* (%)	3 (18.8%)	14 (17.7%)	>0.999
Previous abdominal surgery, *n* (%)	1 (6.2%)	15 (19.0%)	0.381
Tumor distance from anal verge (cm), median (IQR)	8.0 (4.8–10.0)	7.0 (4.0–10.0)	0.841
Neoadjuvant treatment (RT/CRT), *n* (%)	5 (31.3%)	32 (40.5%)	0.582
TNM Stage, *n* (%)			0.856
Stage I	5 (31.2%)	27 (34.2%)	
Stage II	3 (18.8%)	17 (21.5%)	
Stage III	8 (50.0%)	35 (44.3%)	

Values are presented as mean ± SD for normally distributed variables (Age), median (IQR) for non-normally distributed variables (BMI, Tumor distance), or number (%) for categorical variables. ASA: American Society of Anesthesiologists; RT: radiotherapy; CRT: chemoradiotherapy. Neoadjuvant treatment includes patients receiving either RT alone (e.g., short-course) or CRT. *p*-values were calculated using the independent *t*-test for continuous variables and Pearson’s chi-square test or Fisher’s exact test for categorical variables. For TNM Stage comparison, the chi-square test was performed on the overall distribution.

**Table 2:** Perioperative Outcomes According to Learning Phase.

Outcome	Phase 1 (Learning) (*n* = 16)	Phase 2 (Proficiency) (*n* = 79)	*p*-Value
Total operative time (min), median (IQR)	327.0 (306.8–356.3)	315.0 (272.5–360.0)	0.429
Console time (min), median (IQR)	135.0 (97.5–160.0)	100.0 (85.0–140.0)	0.057
Docking time (min), median (IQR)	14.5 (11.5–15.2)	10.0 (8.5–11.5)	<0.001 *
Robotic stapler firings (*n*), median (IQR)	2.0 (2.0–2.0)	2.0 (2.0–2.0)	0.974
Stapler cartridge type (45 mm), *n* (%)	16 (100.0%)	69 (87.3%)	0.204
Estimated blood loss (mL), median (IQR)	10.0 (10.0–10.0)	10.0 (10.0–10.0)	0.440
Conversion to open surgery, *n* (%)	0 (0.0%)	0 (0.0%)	>0.999
Time to soft diet (days), median (IQR)	2.5 (2.0–4.2)	3.0 (3.0–3.0)	0.459
Postoperative LOS (days), median (IQR)	5.5 (5.0–6.2)	5.0 (4.0–6.0)	0.036 *
Complications, *n* (%)			
Overall (Clavien-Dindo ≥ I)	0 (0.0%)	11 (13.9%)	0.201
Major (Clavien-Dindo ≥ III)	0 (0.0%)	2 (2.5%)	>0.999
Anastomotic leakage	0 (0.0%)	2 (2.5%)	>0.999

Values are presented as median (IQR) for continuous variables, or number (percentage) for categorical variables. *p*-values were calculated using the Mann–Whitney U test for continuous variables and Fisher’s exact test for categorical variables. * Indicates statistical significance (*p* < 0.05). The number of robotic stapler firings refers to the total number of cartridges used for rectal transection. Stapler cartridge type refers to the length of the da Vinci SureForm™ Stapler used (45 mm vs. 60 mm). The percentage represents the proportion of cases where a 45 mm cartridge was the primary stapler used. Two cases in Phase 2 where transection was performed using other methods (e.g., TA stapler or hand-sewn) were excluded from this specific percentage calculation.

**Table 3:** Pathological and Oncological Outcomes.

Pathological Outcome	Phase 1 (Learning) (*n* = 16)	Phase 2 (Proficiency) (*n* = 79)	*p*-Value
Lymph node yield (*n*), median (IQR)	22.0 (18.5–27.2)	20.0 (16.0–26.0)	0.269
DRM (cm), median (IQR)	1.6 (1.0–2.0)	2.5 (1.5–4.0)	0.010 *
CRM (mm), median (IQR)	22.5 (10.0–35.0)	20.0 (10.0–30.0)	0.842
Positive CRM (<1 mm), *n* (%)	1 (6.2%)	3 (3.8%)	0.528
Positive distal margin, *n* (%)	0 (0.0%)	0 (0.0%)	>0.999
TNM Stage, *n* (%)			
Stage I	7 (43.8%)	25 (31.6%)	0.519
Stage II	2 (12.5%)	18 (22.8%)	0.509
Stage III	7 (43.8%)	36 (45.6%)	>0.999

Values are presented as median (interquartile range, IQR) for continuous variables, or number (percentage) for categorical variables. *p*-values were calculated using the Mann–Whitney U test or Fisher’s exact test. * indicates statistical significance (*p* < 0.05).

## Data Availability

The data presented in this study are available on request from the corresponding author. The data are not publicly available due to privacy and ethical restrictions.

## Supplementary Materials

The following supporting information can be downloaded at: https://www.mdpi.com/article/10.3390/jcm15031248/s1, Figure S1: port placement.

## Abbreviations

The following abbreviations are used in this manuscript:

LAR	Low Anterior Resection
TME	Total Mesorectal Excision
CUSUM	Cumulative Sum
ERAS	Enhanced Recovery After Surgery
CRM	Circumferential Resection Margin
DRM	Distal Resection Margin
LOS	Length of Stay
BMI	Body Mass Index
ASA	American Society of Anesthesiologists
nCRT	Neoadjuvant Chemoradiotherapy
SD	Standard Deviation
