# Supplementary figures and images for: Learning Curve of Da Vinci Xi Robotic Low Anterior Resection: A Cumulative Sum Analysis of a Single High-Volume Surgeon
Source: J Clin Med. 2026 Feb 4;15(3):1248. doi: 10.3390/jcm15031248 (PMC12898540; doi:10.3390/jcm15031248)

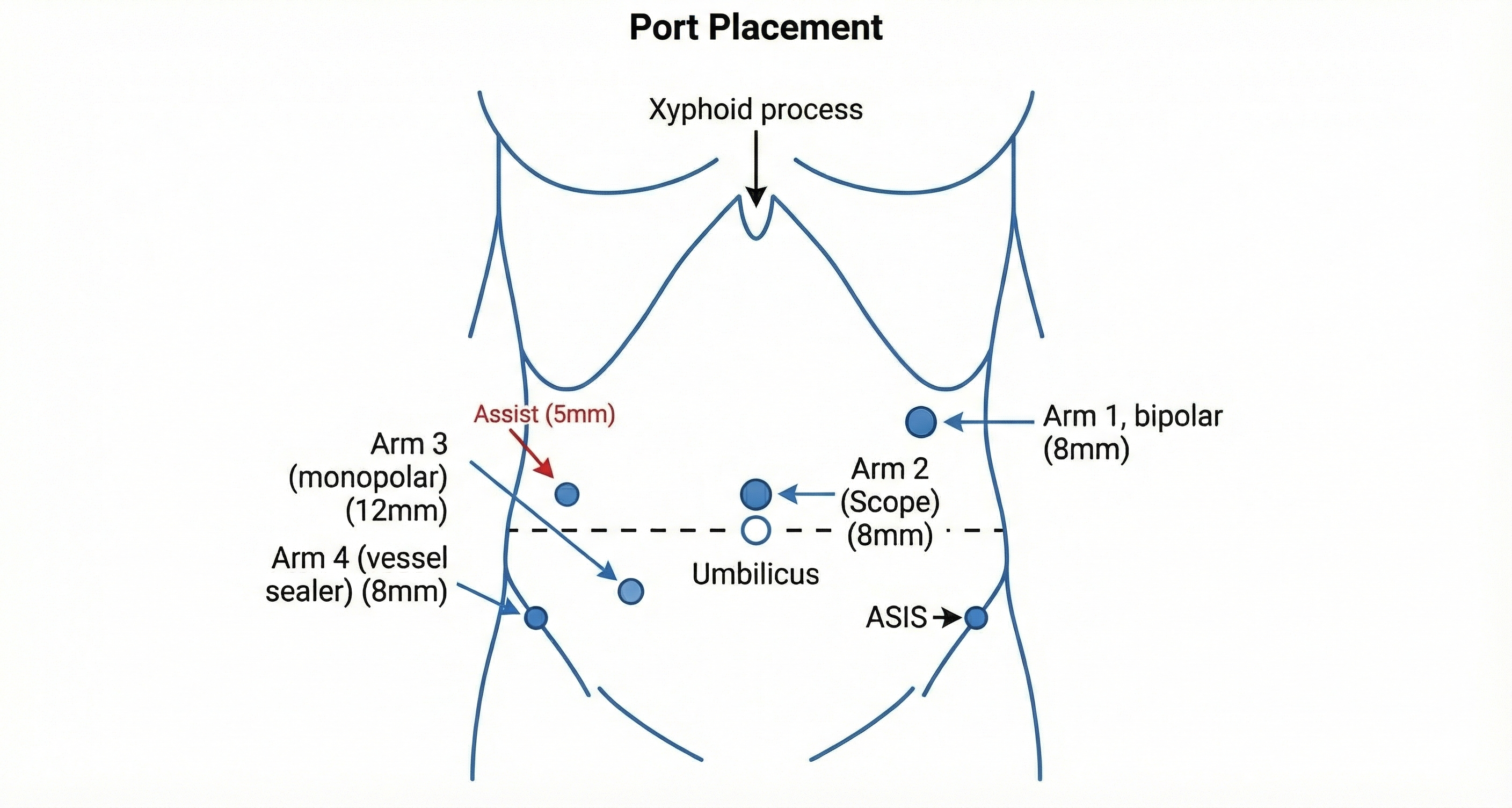

Supplement: Supplementary file 1 [file jcm-15-01248-s001.zip › Figure S1.png]
